# Exploring the genetic architecture of feed efficiency traits in chickens

**DOI:** 10.1038/s41598-021-84125-9

**Published:** 2021-02-25

**Authors:** Jorge Augusto Petroli Marchesi, Rafael Keith Ono, Maurício Egídio Cantão, Adriana Mércia Guaratini Ibelli, Jane de Oliveira Peixoto, Gabriel Costa Monteiro Moreira, Thaís Fernanda Godoy, Luiz Lehmann Coutinho, Danísio Prado Munari, Mônica Corrêa Ledur

**Affiliations:** 1 Faculdade de Ciências Agrárias e Veterinárias, Universidade Estadual Paulista “Júlio de Mesquita Filho”, Jaboticabal, SP 14884-900 Brazil; 2 Embrapa Suínos e Aves, Concórdia, SC 89715-899 Brazil; 3 Departamento de Zootecnia, Escola Superior de Agricultura “Luiz de Queiroz”, Universidade de São Paulo, Av. Pádua Dias 11, Piracicaba, SP 13419-900 Brazil; 4 Present Address: Departamento de Genética, Universidade de São Paulo, Ribeirão Preto, SP 14049-900 Brazil; 5 Present Address: Pamplona Alimentos S/A, Rio do Sul, SC 89164-900 Brazil

**Keywords:** Animal breeding, Genomics

## Abstract

Chicken feed efficiency (FE) traits are the most important economic traits in broiler production. Several studies evaluating genetic factors affecting food consumption in chickens are available. However, most of these studies identified genomic regions containing putative quantitative trait *loci* for each trait separately. It is still a challenge to find common gene networks related to these traits. Therefore, here, a genome-wide association study (GWAS) was conducted to explore candidate genomic regions responsible for Feed Intake (FI), Body Weight Gain (BWG) and Feed Conversion Ratio (FCR) traits and their gene networks. A total of 1430 broilers from an experimental population was genotyped with the high density Affymetrix 600K SNP array. A total of 119 associated SNPs located in 20 chromosomes were identified, where some of them were common in more than one FE trait. In addition, novel genomic regions were prospected considering the SNPs dominance effects and sex interaction, identifying putative candidate genes only when these effects were fit in the model. Relevant candidate genes such as *ATRNL1, PIK3C2A, PTPRN2, SORCS3* and *gga-mir-1759* were highlighted in this study helping to elucidate the genomic architecture of feed efficiency traits. These results provide new insights on the mechanisms underlying the consumption and utilization of food in chickens.

## Introduction

A major concern in current animal production is to increase productivity to meet the growing demands for animal protein in a more sustainable and efficient way, minimizing their impact on the environment and natural resources. An effective way to address this challenge is to improve the animal performance in terms of food consumption and its better use^1^. Furthermore, improving grow-finish feed efficiency will reduce production cost and increase profitability. Thus, feed efficiency (FE) is one of the most important selection criteria implemented in breeding programs, playing a decisive role in the economic benefit of animal meat production^2,3^. The FE depends on the relation between input—feed intake (FI) and output—growth or body weight gain (BWG) of an animal, and the feed conversion ratio (FCR) is widely used to measure it^2,4,5^. Broiler chickens are the most efficient among the farm animals in terms of FE and body weight gain (BWG). Over the last few years, the broilers BWG has increased by 30.2 g per year at the same time that the FCR has reduced annually by 0.036%, contributing to an increase of 167% in poultry meat production, responsible for 35% of the total meat production in the last 30 years^6,7^. All this success is due to the efforts of the poultry industry and breeding programs directed to meet market demand^4^.

Understanding the genetic mechanisms underlying quantitative traits can be useful for defining priors for variances in genomic selection and for identifying candidate genes for marker assisted or gene based selection^8^. In the last 20 years, quantitative trait loci (QTL) mapping studies have identified several chromosome regions that influence the phenotypic variability for many economically important traits in chicken^9–14^. Several approaches such as linkage analyses, candidate gene association and gene expression studies have been performed to elucidate the genetic background underlying complex traits^15–18^. For FI, FCR and BWG, 260 QTL have been mapped on various chromosomes (https://www.animalgenome.org/cgi-bin/QTLdb/GG/browse, accessed in November, 2020). However, FE traits have a complex biology and are mediated by a large number of genes acting at the molecular level as signaling molecules^4,5^, thus traditional QTL mapping studies and candidate gene approaches are insufficient to detect all the genetic mechanisms underlying their variation.

In the last decade, the development of chicken SNP panels provided a powerful tool for genome-wide association studies (GWAS) allowing to detect small associated chromosomal regions, providing more precise estimates of the allele effects at the associated *loci* in farm animals. To date, GWAS has been widely used to map an increasing number of QTL, SNPs and candidate regions associated with FI, BWG, and FCR in beef cattle^19,20^ and swine^8,21^. In chickens, several GWAS have revealed many genes and genomic regions associated with production^11,14,22–24^, morphological traits^13,25^, aggressive behavior^10^, disease resistance^26^, and even with the intestinal microbial interaction^27^. Despite advances in knowledge of regions of interest in the chicken genome, few GWAS has been carried out to evaluate the genetic architecture of FE traits, and most of them ignore dominance, epistasis and sex interactions effects. Taken these effects into account may allow the identification of new candidate genes related to FE traits.

Therefore, this study aimed to identify new chromosomal regions influencing FE traits in a paternal broiler line through genome-wide scale analyses using the high density 600K SNP array. A single-marker GWAS was performed to identify genomic regions and candidate genes with additive, additive + dominance, dominance and sex interaction effects associated with FE traits comprised by FCR, FI, and BWG from 35 to 41 days of age.

## Results

### Descriptive statistics and heritability estimates

In the present study, records from approximately 1300 chickens from the broiler population studied were available for the three evaluated traits, with the following means from 35 to 41 days of age: 2.27 for FCR, 1091 g for FI and 489.3 g for BWG (Table 1). Males differed significantly from females for all evaluated FE traits, having higher FI and BWG and better FCR than females, as expected in broiler chickens (Table 1). The heritability estimates for all the evaluated traits were low, indicating a large variation due to the environment or the non-additive effects of the genes (Table 2). The genetic correlation (*rg*) between FCR and BWG was high, negative and favorable. The FI was not correlated with FCR and the *rg* between FI and BWG was not accurate (Table 2). Therefore, when selecting for heavier chickens, those have also a better feed conversion. The phenotypic correlation (*rp*) between FI and BWG was positive, indicating a linear association between both traits, as well as the *rp* between BWG and FCR, which also had a favorable linear association (Table 2).

**Table 1:** Descriptive statistics of feed conversion ratio (FCR), feed intake (FI) and body weight gain (BWG) from 35 to 41 days of age for the broiler population used in this study. *N *number of records. *CV *coefficient of variation. a–b Means in same row with different letters are significantly different (p < 0.001).

Trait	N	Mean (SD)	CV%	Min	Max	Male			Female		
						N	Mean (SD)	CV%	N	Mean (SD)	CV%
FCR	1264	2.27 (0.38)	16.74	1.42	3.62	591	2.19a (0.39)	17.8	673	2.34b (0.37)	15.81
FI (g)	1296	1091 (147.18)	13.49	544	1590	607	1153.75a (139.99)	12,13	689	1034.78b (129.90)	12.55
BWG (g)	1293	489.30 (105.01)	21.46	128	802	601	537.80a (103.76)	19,29	692	447.18b (86.30)	19.29

**Table 2:** Heritability estimates (bold, diagonal), genetic correlations (above diagonal) and phenotypic correlations (below diagonal) for feed conversion ratio (FCR), feed intake (FI) and body weight gain (BWG). Standard errors are in parentheses.

Trait	FCR	FI	BWG
FCR	**0.181 **_**(0.056)**_	0.249 _(0.245)_	− 0.797 _(0.094)_
FI	0.023 _(0.031)_	**0.130 **_**(0.047)**_	0.360 _(0.225)_
BWG	− 0.790 _(0.012)_	0.562 _(0.021)_	**0.133 **_**(0.048)**_

### Genome-wide association analysis

In the quality control, 70,552 SNPs failed in the call rate, 121,199 failed the MAF criteria and 13,434 were excluded based on the HWE test. The 15,305 SNPs located in the sex chromosome Z were included in the analysis, as well as the 4791 SNPs with undefined positions, which were assigned as located in chromosome 0. From the 1430 genotyped samples, 22 were removed in the quality control. The parental generation, which was used for pedigree checking, and the offspring without records for the evaluated traits were also removed from further analysis. Hence, after the quality control, a total of 375,776 SNPs and approximately 1300 samples remained for the final association analysis (Supplementary Table S1). The MDS analysis indicated the absence of population stratification in the studied population (Supplementary Fig. S1). The QQ plots and their respective inflation factors (lambda) are shown in Supplementary Fig. S2.

By performing the genome-wide association analyses, 119 SNPs in 20 different *G. gallus* chromosomes (GGA) were identified with all levels of significance for FE traits considering the different SNP effects fit in the model (Supplementary Table S2). From those, only 7 SNPs were significant at 5% genome-wide, while 29 SNPs had moderate association with the studied FE traits, some of them were associated with more than one SNP effect fit in the model (Table 3). The 119 associated SNPs revealed 105 putative candidate genes regulating the FE traits (Supplementary Table S2).

**Table 3:** SNPs with 5% genome-wide significance (in bold) and with moderate association with body weight gain (BWG), feed conversion ratio (FCR) and feed intake (FI) traits identified with different SNP effects fit in the model.

Trait	SNP effect	SNP ID	GGA	Position (bp)	*P *value
BWG	A	rs316558717	5	38,947,151	5.51e−06
BWG	A	rs14992480	13	3,320,197	6.86e−06
**BWG**	**A_sex**	**rs317588919**	**6**	28,493,301	**7.20e**−**07**
BWG	A + D_sex	rs318192494	2	62,758,807	9.74e−06
BWG	A + D_sex	rs312928242	12	8,058,572	5.02e−06
FCR	A	rs13853250	1	38,041,526	5.63e−06
FCR	A_sex	rs314388497	6	29,216,961	9.29e−06
FCR	A_sex	rs15936523	8	26,493,993	8.54e−06
FCR	A_sex	rs314893988	8	25,324,177	8.69e−06
**FCR**	**A + D**	**rs318126183**	**4**	46,851,357	**1.16e**−**07**
FCR	A + D	rs316219360	1	195,411,003	5.89e−06
FCR	A + D	rs316420587	1	194,786,435	7.07e−06
FCR	A + D	rs14513380	5	9,756,115	5.8e−06
FCR	A + D	rs314727166	11	3,384,475	4.86e−06
FCR	A + D_sex	rs312879906	2	76,462,592	2.08e−06
FCR	A + D_sex	rs313006651	4	5,995,991	4.61e−06
FCR	A + D_sex	rs13642049	4	5,496,189	5.77e−06
FCR	A + D_sex	rs15936573	8	26,507,284	7.19e−06
FCR	A + D_sex	rs313312956	12	8,077,730	7.29e−06
**FCR**	**D**	**rs318126183**	**4**	46,851,357	**6.04e**−**08**
**FCR**	**D**	**rs314727166**	**11**	3,384,475	**1.35e**−**06**
FCR	D	rs314487130	1	194,771,508	5.27e−06
FCR	D	rs316219360	1	195,411,003	6.28e−06
FCR	D	rs14325026	3	21,872,815	9.55e−06
FCR	D	rs314377290	4	35,113,081	6.98e−06
FI	A	rs313589731	1	27,315,930	8.50e−06
FI	A	rs312763768	1	102,001,483	9.50e−06
FI	A	rs316682850	7	11,676,924	8.46e−06
FI	A	rs317640967	21	5,762,426	7.63e−06
**FI**	**A_sex**	**rs317066057**	**7**	16,224,389	**7.16e**−**07**
FI	A_sex	rs317640967	21	5,762,426	7.08e−06
**FI**	**A + D**	**rs14845471**	**1**	77,114,645	**1.20e**−**06**
**FI**	**A + D**	**rs15384208**	**1**	110,917,887	**1.63e**−**06**
FI	A + D	rs14846017	1	77,666,765	2.19e−06
FI	A + D	rs315107540	1	112,553,707	4.22e−06
FI	A + D	rs313589731	1	27,315,930	8.5e−06
FI	A + D	rs312763768	1	102,001,483	9.5e−06
FI	A + D	rs14069483	14	1,426,739	3.28e−06
FI	A + D_sex	rs14846017	1	77,666,765	6.16e−06
FI	A + D_sex	rs317066057	7	16,224,389	5.94e−06
FI	A + D_sex	rs314874196	21	362,229	9.20e−06
**FI**	**D**	**rs14069483**	**14**	**1,426,739**	**5.11e**−**07**

### Feed intake (FI)

**Figure 1:**
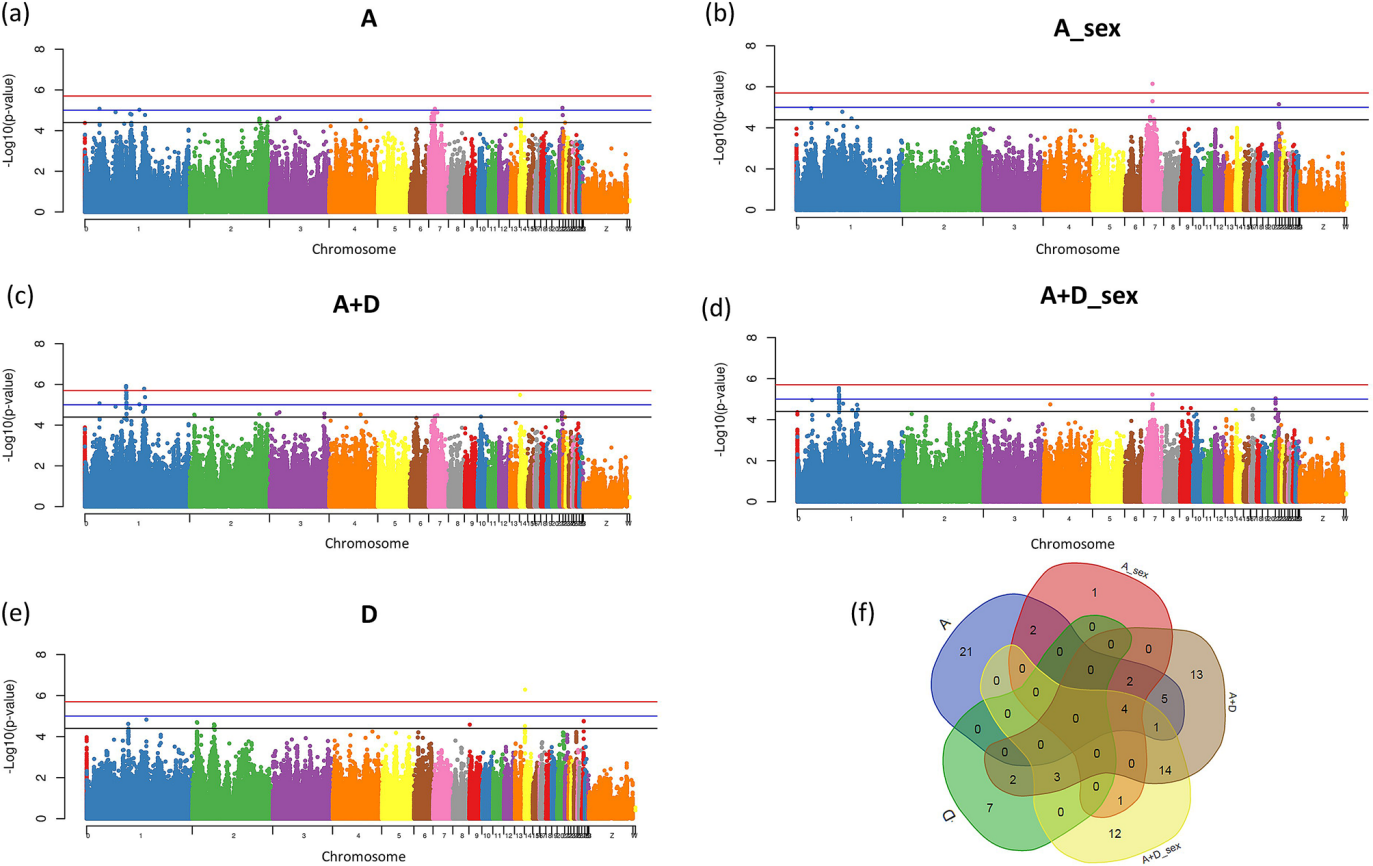
Manhattan plots of genome-wide association study for feed intake (FI). The Manhattan plot indicates the -log10 (p-values) for genome-wide SNPs (y-axis) plotted against their respective positions on each chromosome (x-axis). The horizontal red, blue and black lines indicate the 5% genome-wide significant (4 × 10^–6^), moderate (1 × 10^–5^) and suggestive (4 × 10^–5^) thresholds, respectively. The Manhattan plots are presented fitting five different SNP effects in the model: (**a**) additive effect (A); (**b**) additive fit in each sex (A_sex); (**c**) additive plus dominance (A + D); (**d**) additive plus dominance fit in each sex (A + D_sex); (**e**) dominance (D). The (**f**) is a Venn diagram summarizing the number of significant SNPs identified when each different SNP effect was fit in the model. The Venn diagram was constructed using a Bioinformatics and Evolutionary Genomics tool available in http://bioinformatics.psb.ugent.be/webtools/Venn/.

We observed a total of 47 SNPs associated with FI in 13 different chromosomes with the different SNP effects fit in the model (Fig. 1a–e, Supplementary Table S2). Of those, 4 were significantly associated at 5% genome-wide, 9 SNPs had moderate association and 44 SNPs had suggestive association, some of them were associated with more than one SNP effect fit in the model. Using the A + D SNP effect in the model, two significant SNPs, the rs14845471 and rs15384208, were identified in GGA 1 (Fig. 1c). The SNP rs14845471 is located near the *Zinc Finger Protein 384* (*ZNF384*) gene. When the SNP additive (A) effect was fit within each sex separately (A_sex) in the model, one significant SNP was identified in GGA 7 (rs317066057) (Fig. 1b) and with the SNP dominance (D) effect fit in the model, another SNP was detected in GGA 14 (rs14069483) (Fig. 1e). Many identified SNPs were unique to each different SNP effect fit in the model, while others were identified with more than one SNP effect fit in the model. The results are summarized in Fig. 1f. Perhaps unexpectedly, we found very few SNPs associated with FI, which had their effects influenced by the sex of the chicken. Out of a total of 4 significantly associated SNPs, only 1 seemed to be caused by SNP × sex interaction captured when the A_sex SNP effect was fit in the model (Fig. 1b). From the 9 SNPs with moderate association, 4 were influenced by sex (Table 3, Fig. 1b,d).

**Figure 2:**
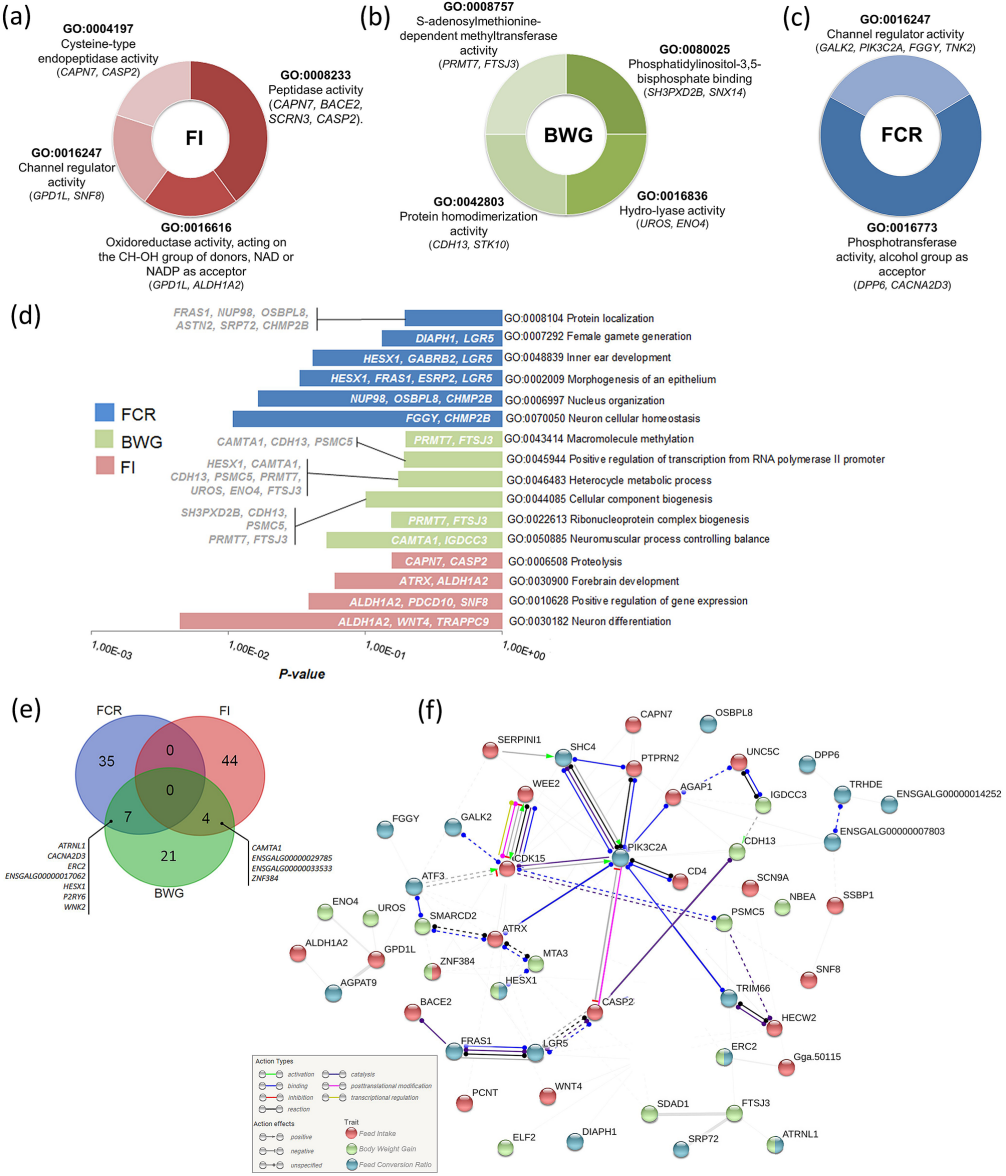
Enrichment analysis and gene networks for the candidate genes identified as associated with feed efficiency (FE) traits. Gene ontology (GO) analysis for terms of (**a**–**c**) molecular function and (**d**) biological processes; (**e**) Venn diagram summarizing the candidate genes found for the FE traits; (**f**) analysis of gene networks of candidate genes identified for feed intake (red nodes), body weight gain (green nodes) and feed conversion ratio (blue nodes). The gene network was constructed using STRING v10^62^.

A total of 35 known genes and 14 uncharacterized genes were found associated with FI (Supplementary Table S2). The Fig. 2a–f shows the main gene ontologies and networks. Two microRNAs (miRNA) were also identified associated with FI, gga-mir-6672 and gga-mir-1641. The genes identified in the functional analysis are mainly related to the activity of peptidases such as Cysteine and channel regulation activity (Fig. 2a), and are involved in biological processes related to forebrain development and neuron differentiation (Fig. 2d).

### Body weight gain (BWG)

**Figure 3:**
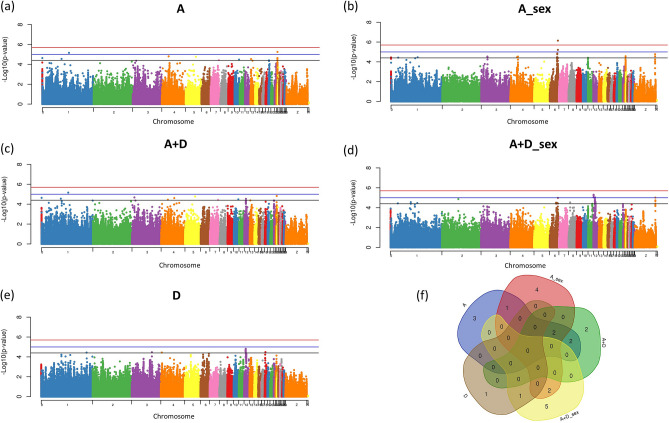
Manhattan plots of genome-wide association study for body weight gain (BWG). The Manhattan plot indicates the −log10 (p-values) for genome-wide SNPs (y-axis) plotted against their respective positions on each chromosome (x-axis). The horizontal red, blue and black lines indicate the 5% genome-wide significant (4 × 10^–6^), moderate (1 × 10^–5^) and suggestive (4 × 10^–5^) thresholds, respectively. The Manhattan plots are presented fitting five different SNP effects in the model: (**a**) additive effect (A); (**b**) additive fit in each sex (A_sex); (**c**) additive plus dominance (A + D); (**d**) additive plus dominance fit in each sex (A + D_sex); (**e**) dominance (D). The (**f**) is a Venn diagram summarizing the number of significant SNPs identified when each different SNP effect was fit in the model. The Venn diagram was constructed using a Bioinformatics and Evolutionary Genomics tool available in http://bioinformatics.psb.ugent.be/webtools/Venn/.

For BWG, a significantly associated SNP, 4 SNPs with moderate association and 19 SNPs with suggestive association were found in 16 chromosomes with the different SNP effects fit in the model (Fig. 3a–e; Supplementary Table S2). Only one significantly associated SNP (rs317588919) was identified using the A_sex effect and it is located in GGA 6 near the *Attractin Like 1* (*ATRNL1*) candidate gene (Fig. 3b). Also, the model considering the SNP effect fit separately in each sex, A_sex and A + D_sex, revealed several SNPs with suggestive association with BWG on the sex chromosome Z (Fig. 3b,d). The number of common and specific SNPs identified with each different SNP effect fit in the model is shown in Fig. 3f.

The gga-mir-1759 miRNA was identified as associated with BWG using the A + D_sex SNP effect in the model. Near the SNPs associated with BWG, 25 positional candidate genes were found, of which 4 were also associated with FI: the Calmodulin Binding Transcription Activator 1 (*CAMTA1*), *ENSGALG00000029785*, *ENSGALG00000033533* and *ZNF384* genes (Fig. 2e). The *CAMTA1* gene was enriched in the biological process of neuromuscular process controlling balance, heterocycle metabolic process and positive regulation of transcription (Fig. 2d). The major molecular functions enriched with the BWG genes are involved with proteolytic functions (Fig. 2b).

### Feed conversion ratio (FCR)

**Figure 4:**
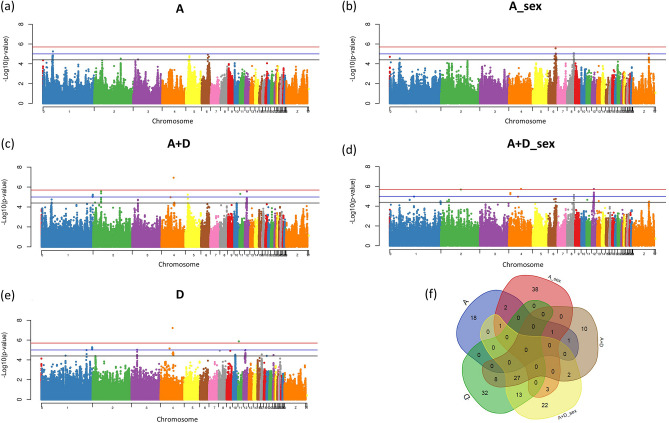
Manhattan plots of genome-wide association study for feed conversion ratio (FCR). The Manhattan plot indicates the -log10 (p-values) for genome-wide SNPs (y-axis) plotted against their respective positions on each chromosome (x-axis). The horizontal red, blue and black lines indicate the 5% genome-wide significant (4 × 10^–6^), moderate (1 × 10^–5^) and suggestive (4 × 10^–5^) thresholds, respectively. The Manhattan plots are presented fitting five different SNP effects in the model: (**a**) additive effect (A); (**b**) additive fit in each sex (A_sex); (**c**) additive plus dominance (A + D); (**d**) additive plus dominance fit in each sex (A + D_sex); (**e**) dominance (D). The (**f**) is a Venn diagram summarizing the number of significant SNPs identified when each different SNP effect was fit in the model. The Venn diagram was constructed using a Bioinformatics and Evolutionary Genomics tool available in http://bioinformatics.psb.ugent.be/webtools/Venn/.

In the present study, 2 SNPs significantly associated, 16 SNPs with moderate association (Table 3) and 42 suggestively associated with FCR were found in 17 chromosomes when the different SNP effects were fit in the model (Supplementary Table S2 and Fig. 4a–e). The rs318126183 was the most significant SNP for FCR identified with the A + D (p = 1.16e−07) and D (p = 6.04e−08) SNP effects fit in the model, having a moderate association with the A + D_sex SNP effect (p = 2.24e−05). This SNP is located next to the uncharacterized gene *ENSGALG00000011221* (Supplementary Table S2). For FCR, the main enriched molecular functions for the candidate genes identified were related to the regulator activity of channels and phosphotransferase (Fig. 2c). Two miRNAs, gga-mir-1730 and gga-mir-1744, were associated with FCR trait. The genes identified were enriched in 6 biological processes involved with morphogenesis of an epithelium and neuron cellular homeostasis (Fig. 2d). The phosphatidylinositol-4-phosphate 3-kinase C2 domain-containing alpha polypeptide (*PIK3C2A*) gene that was associated with this trait appears to interact with several other genes identified as regulating FCR and FI in the gene network analysis (Fig. 2f).

## Discussion

In this study, the heritability estimates for all the FE traits were low. This could possibly be due to the short period that the records were measured. Individual FCR is a difficult and expensive trait to measure in poultry breeding programs, because it needs the evaluation of individual records of feed intake in addition to body weight gain. Therefore, a short evaluated period is commonly used comprising the pre-slaughtered period. Nevertheless, these traits remain as important selection criterion in breeding programs because feed costs represent about 70% of the total cost of the broiler production. According to some authors^28,29^, in broilers, information on FCR is abundant possibly due to the producers' direct association of costs and profits to the amount of feed. In the current study, the lowest heritability estimate found was for FI (0.13 ± 0.047), which was lower than that reported by Gaya et al.^29^ (0.20 ± 0.03). The heritability estimate for BWG (0.133 ± 0.048) was similar to those reported by Pakdel et al.^30^ (0.16 ± 0.05). Furthermore, the heritability estimate of FCR (0.18 ± 0.056) was similar to that reported by Gaya et al.^29^ (0.16 ± 0.03) evaluated from 35 to 49 and Argentão et al.^31^ (0.19) from 35 to 42 days of age. Cruz et al.^32^ also observed low magnitude of the heritability estimates of FCR (0.09 ± 0.04), BWG (0.10 ± 0.04) and FI (0.18 ± 0.05) from 35 to 41 days of age when evaluating these and other performance and carcass traits in the same paternal broiler line used in the present study. Analyzing the genomic heritability of an F_2_ Chicken Resource Population, Moreira et al.^12^ observed lower estimates (from 0.0002 ± 0.0002 to 0.07 ± 0.0143) than those found in the current study for FCR, BWG, and FI.

Feed efficiency is a complex trait affected by feed and growth factors. FCR, defined as the amount of feed consumed per unit of weight gain, is a trait composed by the initial and final body weights and FI. Based on the heritability and genetic correlation estimates, selection for low FCR will improve FE with an expected correlated response in increased BWG. The positive genetic correlation between FI and BWG indicates that fast growing broilers have been consuming increased amount of feed due to their greater appetite, as also shown by Aggrey et al.^28^ comparing residual feed intake and BWG.

The genetic correlation found for FI and FCR, considering the period from 35 to 41 days, were different than those found for these traits by other authors^28,33^, but similar to that found by Cruz et al.^32^ (0.39 ± 0.23). These differences may be due to the nature of the pleiotropic relationship between FI and FCR, which may be dependent on age. Consequently, the molecular, physiological and nutritional factors that control FI and FCR may also depend on the time of development^2^. As with other studies^28,33^, the correlations of both FI and FCR cannot be predicted accurately because of the inherent problem of FCR being a ratio trait^28^.

The poultry geneticists have used GWAS extensively as a powerful tool for elucidating the genetic mechanisms that determine FE. In recent years, several studies have identified different QTLs controlling traits in chickens such as FI^4,5,12,14,34^, FCR^4,5,12,14,34,35^, and Body Weight or Weight Gain^4,5,9,12^. However, many of the differences between screened loci that putatively influence FE are breed-, age-, or breeding area-specific^34^. In our study, new regions and candidate genes not yet described were identified, demonstrating the complexity and the large number of biological pathways involved in the regulation of these traits. In addition, most of the studies used approaches to identify only the additive genetic variation, due to their applicability in breeding programs, forgetting the other forms of gene interaction that regulate these traits, such as dominance and sex interaction effects which are addressed in our study. According to Hayes et al.^36^, when non-additive effects are considered in the model, predictions could be improved, because the genetic architecture of traits contributes to the accuracy of models. With the emergence of new technologies such as gene editing, the identification of genes with non-additive genetic influence on the regulation of FE traits may now have an application in animal breeding programs of the future^37^. To our knowledge, the study presented here is the first GWAS exploring different genetic effects in feed efficiency traits in chickens with the 600K SNP array.

In the present study, several SNPs with dominance effect related to FI, BWG and FCR were identified. Among these markers, two had significant dominance effects for FCR (Fig. 4e), and one showed significant dominance effect for FI (Fig. 1e), while the rest of the SNPs had only moderate or suggestive association. Although this number is low, these results show convincing evidence that dominance has an important role in the genetic control of FE traits. Our results also suggest that additive effects contribute more to feed efficiency genetic variation than do dominance effects and are in agreement with other studies. Li et al.^38^ showed that additive model and the combined additive and dominance models produced the majority of significant SNPs in feed-related traits. Recently, Darwish et al.^39^ demonstrated for eggshell blueness chickens that the contribution of genotypic effects of some SNPs were further divided into additive and dominant effects depending on the trait.

Our results have shown that additive or additive + dominance effects of several SNPs were influenced by the sex of the chicken, highlighting their importance in FE traits, mainly for BWG and FCR (Figs. 3f, 4f). When the SNP effects were fit separately in each sex, novel genomic regions were prospected. Moreover, putative candidate genes, such as the Sortilin Related VPS10 Domain Containing Receptor 3 (*SORCS3*), were identified only when this effect was taken into account. The *SORCS3* gene encodes a type-I receptor transmembrane protein that is expressed in neurons of the arcuate nucleus of the hypothalamus and regulate biological processes such as control energy balance and orexigenic peptide production. A study in mice showed that combined deficiency of SORCS3 and SORCS1 may result in a state of chronic energy excess^40^.

Several biological processes related to the development and functioning of the brain were enriched with the candidate genes identified in the GWAS for these traits (Fig. 2d). For FCR, genes involved with Neuron cellular homeostasis (GO:0070050) were observed, while genes that act on the Forebrain development (GO:0030900) and Neuron differentiation (GO:0030182) were identified for FI. One of the genes enriched for the Neuron differentiation is the *Trafficking Protein Particle Complex 9* (*TRAPPC*9) that may function in neuronal cells differentiation and has been associated with diseases related to Intellectual Disability-Obesity-Brain in humans^41^. It is already known that FI and energy metabolism regulate the energy balance and expenditure, as well as the hormone secretion, where the brain has the important role of inducing and maintaining the homeostasis ^42^.

An interesting candidate gene revealed from the GWAS for FCR was the *PIK3C2A*. This gene showed several potential interactions with other genes associated with FCR and FI (Fig. 2f) and seems to be an important regulator of biological processes common to these traits. The protein encoded by this gene acts in signaling pathways involved in cell proliferation, cell survival, cell migration, intracellular protein trafficking and has an important function in insulin signaling and secretion. *PIK3C2A* is involved with insulin secretion, which is regulated by the insulin receptor and it also participates in the exocytosis of insulin granules. In birds, the regulation of circulating levels of insulin and its involvement with the adipose tissue deposition is not completely understood, and it is still not known if this regulation is similar as it is in mammals^43^. However, it was observed that fed or feed-deprived chickens with lesions of the ventromedial hypothalamus present elevated circulating insulin levels, which could led to the development of metabolic obesity^44^.

Another study found that the *PIK3C2A* gene affects the pathway of leptin hormone^45^. The leptin gene was recently identified in chickens^46^, and it is responsible for informing to the hypothalamus that appetite should be inhibited. Alliouachene et al.^45^ observed that when *PIK3C2A* gene was inactivated, male mice showed early onset of leptin resistance and also a defect in leptin signaling in the hypothalamus leading to a mild, age-dependent obesity, insulin resistance and glucose intolerance.

One of the predicted interactions of the *PIK3C2A* gene was with the *Protein Tyrosine Phosphatase, Receptor Type N2* (*PTPRN2*) gene that has been associated with FI (Fig. 2f). The *PTPRSN2* gene also plays a role in insulin secretion in response to glucose stimuli and is required for normal accumulation of the neurotransmitters norepinephrine, dopamine and serotonin in the brain. Reyer et al.^4^, and later Mebratie et al.^5^, searching this data with a mixed linear model approach, also identified genes from the *Protein tyrosine phosphatases family, receptor type* (*PTPR*) associated with feed conversion efficiency traits in a commercial broiler line. Both the *PTPRG* and *PTPRC* genes were identified as possible controllers of body weight traits (36 and 46 days).

Moreover, some candidate genes were associated with more than one trait in our study, evidencing pleiotropic effect regulating feed efficiency in chickens. Among the genes identified in common between BWG and FCR (Fig. 2e), we highlight the *Sortilin Related VPS10 Domain Containing Receptor 3* gene (*SORCS3*). This gene is involved in trafficking the tropomyosin-related kinase B receptor (TrkB) attenuating the brain-derived neurotrophic factor, which plays an important function in the energy homeostasis. It has been demonstrated that the loss in the *SORCS1* and *SORCS3* joint actions led to a chronic energy excess state, which is characterized by enhancing food intake and adiposity, and decreasing locomotor activity, within other characteristics^40^.

Like the *SORCS3* gene, the genes *PDZD8*^47^, *ERC2*^48^ and *ATRNL1* were also identified for both BWG and FCR (Fig. 2e). The first two genes are described as acting in the central nervous system, and the *ATRNL1* plays a role in signaling by central melanocortin receptors and is involved in energy homeostasis^49^. It has been suggested that melanocyte stimulating hormone acting through the melanocortin receptors serves as an important central mediator for leptin action on FI and energy expenditure^50^.

Previous studies have confirmed the contribution of post-transcriptional regulation on the variability of phenotypic traits in broilers^51^. In the current study, five potential miRNAs were identified as candidates to regulate FE traits. The gga-mir-1641, identified for FI, has already been described in ovarian follicles as differentially expressed between low‐ and high‐rate egg production chickens^51^. The miRNA gga-mir-1759, associated here with BWG, has previously been described as a potential regulator of the *Lipin-1*, an important gene involved in triglyceride synthesis and adipocyte differentiation^52^.

The vast majority of the genes identified for all FE traits appear to be somehow involved in the energy homeostasis. At the molecular level, several signaling molecules within the bilateral gut-brain axis contribute to the regulation of food intake and nutrient metabolism^4^. The interrelationship processes between FI and energy homeostasis affect the complex biology of the FCR and consequently the BWG. These signals are integrated by peripheral nerves and brain centers, such as hypothalamus and brain stem, and regulate central neuropeptides modulating feeding and energy expenditure^53^. Our results suggest that non-additive effects are important to the genetic architecture of FE. Dominance effects play a relevant role, mainly for BWG and FCR (Figs. 3f, 4f). The understanding of the genetic interaction of these FE traits brings a new vision of their genetic regulation and the involved biological pathways, making it possible to develop new approaches for breeding programs in the future through the selection of the most relevant target genes.

In summary, this study provides novel insights into the genetics of feed efficiency traits, revealing possible genetic architecture and potential biological pathways interaction between FI, BWG and FCR traits in chickens. A total of 33 genomic regions in 13 chromosomes, containing 41 candidate genes, were found associated with FE traits at 5% significant and moderate genome-wide levels. Key positional candidate genes have been found: *ATRNL1, PIK3C2A, PTPRN2, SORCS3* and *gga-mir-1759*.

The results also suggest pleiotropic effects of these QTL and contribute to our understanding of the genetic basis of FI, BWG and FCR traits in chickens. Therefore, this study offers important knowledge of the potential candidate genes to improve the accuracy of early selection of birds with best feed efficiency traits in the future.

## Material and methods

### Chicken population and phenotype measurement

The chickens used in this study were from an experimental population (TT), generated from the expansion of a paternal broiler pure line TT, developed for genomics studies by the Embrapa Swine and Poultry National Research Center. For the expansion of this line, 20 males were mated with 92 unrelated females (1:5) to generate approximately 1500 chicks from five incubations, resulting in approximately half of each sex. A complete pedigree with 18 generations from 1991 to 2008, containing a total of 2139 individuals was used. The chicks were identified with metallic rings at birth for pedigree control and raised in collective boxes until 35 days of age according to the procedures for commercial broilers. For the evaluation of individual FE traits, chickens were transferred to individual feed cages, where they remained from 35 to 41 days of age. The chickens had free access to water and a corn and soybean meal-based diet throughout the experimental period. Broiler diet containing 3150 kcal/kg of metabolizable energy (ME) and 21% of crude protein (CP) was provided from 1 to 21 days, 3200 kcal/kg ME and 20% CP from 22 to 35 days, and 3200 kcal/kg ME and 18.5% CP from 36 to 41 days of age. Previous genomic studies have been performed in this population, and more details can be found in Marchesi et al.^54^.

Recorded phenotypic data were individual start and final weights (BW35 and BW41), total FI, and total BWG from 35 to 41 days of age. The FCR was calculated as the ratio of FI and BWG. The removal of discrepant values and the descriptive statistics of the phenotype file were conducted in the R software^55^.

### DNA extraction, genotyping and quality control

The extraction of the genomic DNA was performed using PureLink Genomic DNA (Invitrogen, Carlsbad, CA, USA) kit. The extracted DNA was quantified using a Qubit 2.0 Fluorometer (Thermo Fisher Scientific, Waltham, MA, USA) and diluted to a concentration of 10 ng/μL.

Genomic DNA samples from 1430 chickens (652 males and 778 females), of which 113 (20 sires and 93 dams) are the progenitors (born in 2007) and the remaining are the offspring (born in 2008), were genotyped with the 600K Affymetrix Axiom high-density (HD) chicken genotyping array^56^ in the “Centro de Genômica Funcional, ESALQ”, University of São Paulo, Brazil. The genotypes were initially read and edited using the Axiom Analysis Suite from Affymetrix with a DishQC removal value of 0.82. In this study, the data quality control was performed using PLINK v1.07 software^57^, excluding SNPs with call rate below 98%, with significant deviations (p < 10^–6^) from the Hardy–Weinberg equilibrium, and with minor allele frequencies (MAF) lower than 2%. Samples that presented call rates lower than 90% were also excluded. The classical multidimensional scaling (MDS) analysis was used to detect population structure in PLINK v1.07 software^57^.

### Genetic and phenotypic parameter estimates

The environmental effects considered in the genetic analysis model were studied through the least square method. The group effect (sex and hatch) and the linear effect of the covariate body weight at 35 days of age (BW35) were significant (P < 0.05) regarding the traits related to FE from 35 to 41 days of age.

Genetic parameter estimates for the traits studied were performed using the restricted maximum likelihood method (REML) in a multitrait animal model using the WOMBAT software described by Meyer^58^. The animal model proposed for the multitrait analysis was:

1 $$y = Xb + Za + e$$

where *y* is the vector of the dependent variable; *X* is the incidence matrix for fixed effects, correlating elements from *b* and *y*; *b* is the fixed effects vector containing the groups of animals from the same incubations (1 to 5) and sex (1 or 2), and the linear covariate BW35; *Z* is the incidence matrix for the direct genetic random effect, correlating elements from *a* and *y*; *a* is the random effect vector for the direct additive genetic effect, and *e* is the random residual effects vector. Both $a$ and $e$ are assumed to be normal distributed with mean zero and variance $A\sigma_{a}^{2}$ and $I\sigma_{e}^{2}$, respectively. The $\sigma_{a}^{2}$ is the additive genetic and $\sigma_{e}^{2}$ is the error variances, $A$ is the numerator relationship matrix constructed with the pedigree information and $I$ is an identity matrix with order equals to the number of observations.

### Genome-wide association analysis

The linkage disequilibrium (LD) between SNP pairs with a maximum distance of 5 Mb were measured by r^2^ values for the TT population for each chromosome using PLINK v1.07 software^57^.

Genome-wide association analysis using single marker information was performed for FI, BWG, and FCR using the Qxpak 5.0 software^59^, which employs a maximum likelihood approach to test the association between SNP and trait, performed one SNP at a time. Qxpak allows both the additive and dominance fixed effects of the SNP to be fitted in the model. Thus, the GWAS was carried out according to the analysis procedure described by Bolormaa et al.^60^ and Li et al.^38^, based on the following linear mixed model:

2 $$y = Xb + w1_{i} \propto_{i} + w2_{i} \beta_{i} + Za + e$$

where $y$ is the vector of phenotypic records, $X$ is a design matrix, $b$ is a vector of fixed effects consisting of sex, hatch and the covariate BW35, $w1_{i}$ and $w2_{i}$ are vectors containing the additive (− 1, 0 or 1) and dominance (0, 1, 0) genotype codes of each animal at the *i*th SNP, respectively, and fit as covariates, $\propto_{i}$ and $\beta_{i}$ are the scalar regression coefficients for the additive and dominance effects, respectively, $Z$ is an identity matrix, $a$ is a vector of random animal additive (polygenic) effects, and $e$ is a vector of random residual effects. The assumptions for this model were the same as previously described for model 1.

Three separate analyses were performed for each of the 375,776 SNPs: (I) the additive effect of the SNP was fit in the model while the fixed dominance effect $w2_{i} \beta_{i}$ was dropped from the model; (II) the additive and dominance effects of the SNPs were fit in the model and both $w1_{i} \propto_{i}$ and $w2_{i} \beta_{i}$ were retained in the model; and (III) the additive and dominance effects of the SNPs were fit as previously described in (II), but here the significance of the dominance effect was tested after fitting the additive effect.

The hypothesis tests were performed comparing the full model (with the SNP effect as alternative hypothesis) with the reduced model (with no SNP effect as null hypothesis) when the additive and the additive + dominance effects of the SNPs were fit in the model. In addition, we also used the SNP additive effect as reduced model to test for the dominance effect when fitting the additive + dominance effects in the model. Qxpak^59^ computes the likelihood for each SNP and retains the one with the maximum likelihood. The difference in likelihoods between the complete and the reduced model were tested using likelihood ratio test (LRT) with 1 degree of freedom (df) if only the additive effect was fit or 2 df if the dominant effect was also included in the model.

To describe sex differences in the genetic effects and in the amount of variance explained by each SNP, the additive and the additive + dominace effects of the SNPs were fit within each sex separately in model (2), and were denoted as A_sex and A + D_sex. To test the hypothesis of SNP × sex interaction, a reduced model with no SNP effect was compared to the previously mentioned full model.

Since Bonferroni correction is highly conservative due to the high LD in genetic data, it may produce too stringent a threshold and many false negative results. Therefore, we calculated the number of effectively independent SNPs using the—indeppairwise parameter in the PLINK v1.07 software^58^ with a window size of 25 SNPs, a step of 5 SNPs, and an r^2^ threshold of 0.2, resulting in 24,959 independent tests. Therefore, the threshold for the Bonferroni 5% genome-wide significance was a P-value of 2 × 10^–6^ (0.05/24,959). We also used the P-value of 1 × 10^–5^ for a moderate association as recommended by the Wellcome Trust Case and Control Consortium (WTCCC)^61^ and a suggestive association with P-value of 4 × 10^–5^ (1/24,959). Manhattan plots and quantile–quantile plots (QQ-plot) were created using the R software^55^. Inflation factor was also calculated using R.

### Gene identification and enrichment analysis

The SNP positions were updated according to the newest release from Ensembl annotation of the Gallus gallus 5.0 genome version. From the LD data analysis, the SNPs with the lowest p-value in each region in LD (r^2^ ≥ 0.3) with other significant SNPs were identified using a Perl homemade script. The identification of the genes near to significant SNPs was obtained using the Ensembl tool Variant Effect Predictor (VEP) and the UCSC genome browser (https://genome.ucsc.edu/). The classification of genes according to its biological function, identification of metabolic pathways and enrichment of genes was performed using DAVID v. 6.8 (https://david.ncifcrf.gov/tools.jsp), and statistically significant results were considered when false discovery rate (FDR) was lower than 0.20. A gene network of predicted functional proteins was constructed using the STRING 10.0 software^62^.

### Ethics statement

The protocols and the use of animals for this research were approved by the Ethics Committee on Animal Use (CEUA) from the Embrapa Swine and Poultry National Research Center under the protocol # 011/2010.

## Supplementary Information

Supplementary Information.

## Data Availability

The datasets generated during and/or analyzed during the current study are available from the corresponding author on reasonable request.

## References

[1] Hume DA, Whitelaw CBA, Archibald AL (2011). The future of animal production: Improving productivity and sustainability. J. Agric. Sci..

[2] Willems OW, Miller SP, Wood BJ (2013). Aspects of selection for feed efficiency in meat producing poultry. Worlds Poult. Sci. J..

[3] Donohue M, Cunningham DL (2009). Effects of grain and oilseed prices on the costs of US poultry production. J. Appl. Poult. Res..

[4] Reyer H, Hawken R, Murani E, Ponsuksili S, Wimmers K (2015). The genetics of feed conversion efficiency traits in a commercial broiler line. Sci. Rep..

[5] Mebratie W, Reyer H, Wimmers K, Bovenhuis H, Jensen J (2019). Genome wide association study of body weight and feed efficiency traits in a commercial broiler chicken population, a re-visitation. Sci. Rep..

[6] Havenstein G, Ferket P, Qureshi M (2003). Growth, livability, and feed conversion of 1957 versus 2001 broilers when fed representative 1957 and 2001 broiler diets. Poult. Sci..

[7] Hicks TM, Knowles SO, Farouk MM (2018). Global provisioning of red meat for flexitarian diets. Front. Nutr..

[8] Do D (2014). Genome-wide association and systems genetic analyses of residual feed intake, daily feed consumption, backfat and weight gain in pigs. BMC Genet..

[9] Emrani H, Vaez Torshizi R, Akbar Masoudi A, Ehsani A (2017). Identification of new loci for body weight traits in F2 chicken population using genome-wide association study. Livest. Sci..

[10] Li Z (2016). Genome-wide association study of aggressive behaviour in chicken. Sci. Rep..

[11] Moreira GCM (2018). A genome-wide association study reveals novel genomic regions and positional candidate genes for fat deposition in broiler chickens. BMC Genomics.

[12] Moreira GCM (2019). Unraveling genomic associations with feed efficiency and body weight traits in chickens through an integrative approach. BMC Genet..

[13] Shen M (2016). Genome-wide association studies for comb traits in chickens. PLoS ONE.

[14] Yuan J (2015). Genome-wide association studies for feed intake and efficiency in two laying periods of chickens. Genet. Sel. Evol..

[15] Nones K (2006). Mapping QTLs on chicken chromosome 1 for performance and carcass traits in a broiler × layer cross. Anim. Genet..

[16] Abasht B, Dekkers JCM, Lamont SJ (2006). Review of quantitative trait loci identified in the chicken. Poult. Sci..

[17] Huang YQ (2006). Single nucleotide polymorphisms in the chicken Lmbr1 gene are associated with chicken polydactyly. Gene.

[18] Xu HP (2011). Polymorphisms associated with egg number at 300 days of age in chickens. Genet. Mol. Res..

[19] Seabury CM (2017). Genome-wide association study for feed efficiency and growth traits in U.S. beef cattle. BMC Genomics.

[20] de Oliveira PS (2014). Identification of genomic regions associated with feed efficiency in Nelore cattle. BMC Genet..

[21] Ramayo-Caldas Y (2012). Genome-wide association study for intramuscular fatty acid composition in an Iberian × Landrace cross1. J. Anim. Sci..

[22] Gu X (2011). Genome-wide association study of body weight in chicken F2 resource population. PLoS ONE.

[23] Liao R (2016). Genome-wide association study reveals novel variants for growth and egg traits in Dongxiang blue-shelled and White Leghorn chickens. Anim. Genet..

[24] Podisi BK, Knott SA, Burt DW, Hocking PM (2013). Comparative analysis of quantitative trait loci for body weight, growth rate and growth curve parameters from 3 to 72 weeks of age in female chickens of a broiler-layer cross. BMC Genet..

[25] Nie C (2016). Genome-wide association study revealed genomic regions related to white/red earlobe color trait in the Rhode Island Red chickens. BMC Genet..

[26] Wolc A (2013). Genome-wide association study for Marek’s disease mortality in layer chickens. Avian Dis..

[27] Ji J (2019). Association of host genetics with intestinal microbial relevant to body weight in a chicken F2 resource population. Poult. Sci..

[28] Aggrey SE, Karnuah AB, Sebastian B, Anthony NB (2010). Genetic properties of feed efficiency parameters in meat-type chickens. Genet. Sel. Evol..

[29] Gaya LG (2006). Heritability and genetic correlation estimates for performance and carcass and body composition traits in a male broiler line. Poult. Sci..

[30] Pakdel A, Van Arendonk JAM, Vereijken ALJ, Bovenhuis H (2005). Genetic parameters of ascites-related traits in broilers: correlations with feed efficiency and carcase traits. Br. Poult. Sci..

[31] Argentão, C. *et al.* Genetic and phenotypic parameters of growth and carcass traits of a male line of broilers raised in tropical conditions. in *7th World Congress on Genetics Applied to Livestock Production*, 2–5 (2002).

[32] Cruz VAR (2020). Genetic parameters for performance and carcass traits in a paternal 1 lineage of broiler. An. Acad. Bras. Cienc..

[33] Yuan J (2015). Genetic parameters of feed efficiency traits in laying period of chickens. Poult. Sci..

[34] Yuan J (2017). Genome-wide association study reveals putative role of gga-miR-15a in controlling feed conversion ratio in layer chickens. BMC Genomics.

[35] Shah TM (2016). A genome-wide approach to screen for genetic variants in broilers (*Gallus**gallus*) with divergent feed conversion ratio. Mol. Genet. Genomics.

[36] Hayes BJ, Bowman PJ, Chamberlain AJ, Goddard ME (2009). Invited review: Genomic selection in dairy cattle: Progress and challenges. J. Dairy Sci..

[37] Oishi I, Yoshii K, Miyahara D, Kagami H, Tagami T (2016). Targeted mutagenesis in chicken using CRISPR/Cas9 system. Sci. Rep..

[38] Li Y (2017). Evaluation of non-additive genetic variation in feed-related traits of broiler chickens. Poult. Sci..

[39] Darwish HYA (2019). Genome-wide association study and a post replication analysis revealed a promising genomic region and candidate genes for chicken eggshell blueness. PLoS ONE.

[40] Subkhangulova A (2018). sorcs 1 and sorcs 3 control energy balance and orexigenic peptide production. EMBO Rep..

[41] Mochida GH (2009). A truncating mutation of TRAPPC9 is associated with autosomal-recessive intellectual disability and postnatal microcephaly. Am. J. Hum. Genet..

[42] Richards MP, Proszkowiec-Weglarz M (2007). Mechanisms regulating feed intake, energy expenditure, and body weight in poultry. Poult. Sci..

[43] Woods SC, Benoit SC, Clegg DJ (2006). The brain–gut–islet connection. Diabetes.

[44] Sonoda T (1983). Hyperinsulinemia and its role in maintaining the hypothalamic hyperphagia in chickens. Physiol. Behav..

[45] Alliouachene S (2016). Inactivation of class II PI3K-C2α induces leptin resistance, age-dependent insulin resistance and obesity in male mice. Diabetologia.

[46] Farkašová H, Hron T, Pačes J, Pajer P, Elleder D (2016). Identification of a GC-rich leptin gene in chicken. Agri Gene.

[47] Hirabayashi Y (2017). ER-mitochondria tethering by PDZD8 regulates Ca^2+^ dynamics in mammalian neurons. Science.

[48] Wang Y, Liu X, Biederer T, Südhof TC (2002). A family of RIM-binding proteins regulated by alternative splicing: Implications for the genesis of synaptic active zones. Proc. Natl. Acad. Sci. U.S.A..

[49] Walker WP (2007). Genetic analysis of attractin homologs. Genesis.

[50] Forbes S, Bui S, Robinson BR, Hochgeschwender U, Brennan MB (2001). Integrated control of appetite and fat metabolism by the leptin-proopiomelanocortin pathway. Proc. Natl. Acad. Sci..

[51] Liu W (2011). A genome-wide SNP scan reveals novel loci for egg production and quality traits in white leghorn and brown-egg dwarf layers. PLoS ONE.

[52] Zhang SP, Li SY, Chen W, Lu WW, Huang YQ (2013). A single-nucleotide polymorphism in the 3′ untranslated region of the LPIN1 gene and association analysis with performance traits in chicken. Br. Poult. Sci..

[53] Wynne K, Stanley S, McGowan B, Bloom SR (2005). Appetite control. J. Endocrinol..

[54] Marchesi JAP (2018). Relationship of runs of homozygosity with adaptive and production traits in a paternal broiler line. Animal.

[55] R Development Core Team, R. *Computational Many-Particle Physics*. *R Foundation for Statistical Computing*, **739** (Springer, Berlin, 2008).

[56] Kranis A (2013). Development of a high density 600K SNP genotyping array for chicken. BMC Genomics.

[57] Purcell S (2007). PLINK: A tool set for whole-genome association and population-based linkage analyses. Am. J. Hum. Genet..

[58] Meyer K (2007). WOMBAT—A tool for mixed model analyses in quantitative genetics by restricted maximum likelihood (REML). J. Zhejiang Univ. Sci. B.

[59] Pérez-Enciso M, Misztal I (2011). Qxpak.5: Old mixed model solutions for new genomics problems. BMC Bioinform..

[60] Bolormaa S (2015). Non-additive genetic variation in growth, carcass and fertility traits of beef cattle. Genet. Sel. Evol..

[61] Burton PR (2007). Genome-wide association study of 14,000 cases of seven common diseases and 3,000 shared controls. Nature.

[62] Szklarczyk D (2015). STRING v10: Protein–protein interaction networks, integrated over the tree of life. Nucleic Acids Res..

