# Supplementary material for: Exploring the genetic architecture of feed efficiency traits in chickens
Source: Sci Rep. 2021 Feb 25;11:4622. doi: 10.1038/s41598-021-84125-9 (PMC7907133; doi:10.1038/s41598-021-84125-9)
Supplement: Supplementary file 1 — Supplementary Information. [file 41598_2021_84125_MOESM1_ESM.docx]

**Exploring the genetic architecture of feed efficiency traits in chickens**

**Jorge Augusto Petroli Marchesi¹^,^ª, Rafael Keith Ono^2,b^, Mauricio Egidio Cantão^2^, Adriana Mércia Guaratini Ibelli^2^, Jane de Oliveira Peixoto^2^, Gabriel Costa Monteiro Moreira^3^, Thaís Fernanda Godoy^3^, Luiz Lehmann Coutinho^3^, Danísio Prado Munari¹, Mônica Corrêa Ledur^2,*^**

¹Universidade Estadual Paulista “Júlio de Mesquita Filho”, Faculdade de Ciências Agrárias e Veterinárias, São Paulo 14884-900, Brazil.

^2^Embrapa Suínos e Aves, Concórdia, 89715-899 Brazil.

^3^Departamento de Zootecnia, Escola Superior de Agricultura “Luiz de Queiroz”, Universidade de São Paulo, Av. Pádua Dias 11, Piracicaba, São Paulo 13419-900, Brazil.

^a^Present address: Universidade de São Paulo, Departamento de Genética, Ribeirão Preto, 14049-900, Brazil.

^b^Present address: Pamplona Alimentos S/A, Rio do Sul 89164-900, Brazil.

*monica.ledur@embrapa.br


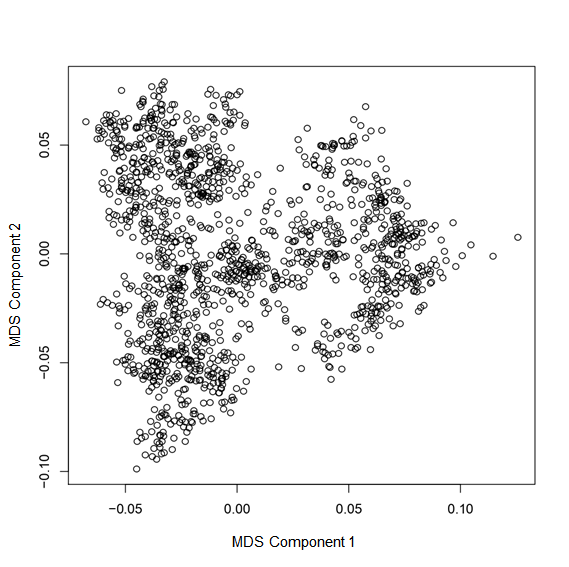


**Supplementary Figure S1.** Multi-dimensional scaling plot to examine for the presence of population stratification.


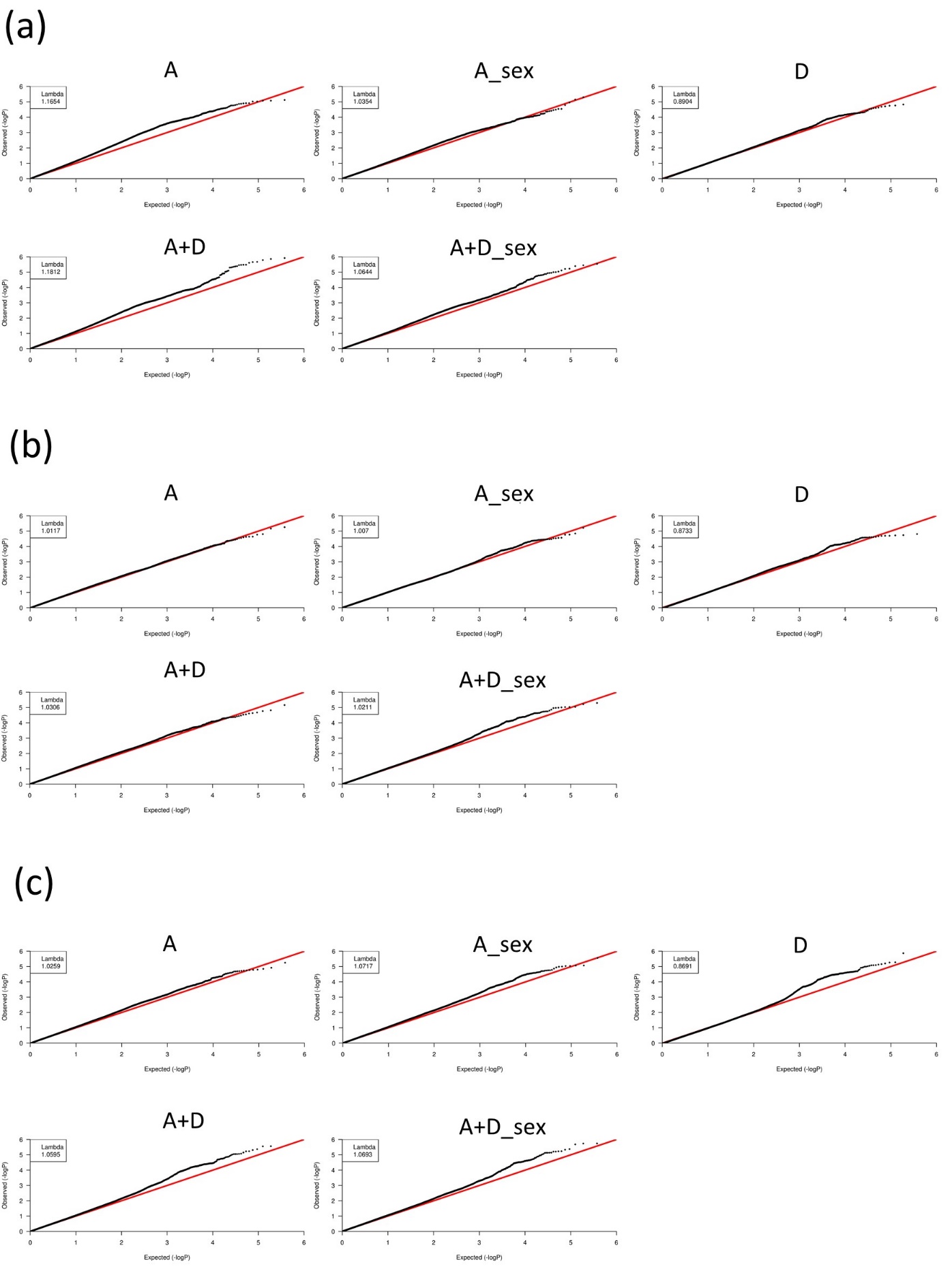
**Supplementary Figure S2.** QQ plot for (a) Feed Intake, (b) Body Weight Gain and (c) Feed Conversion Ratio traits. The x-axis indicates the expected -log10-transformed P-values, and the y-axis shows the observed -log10-transformed P-values. The genomic inflation factors (Lambda) are shown on the top left in the QQ plots.

| **Chromosome** | **Chromosome size (Kb)*** | **Total SNPs** | **SNP density (*n*/Kb)** |
| --- | --- | --- | --- |
| 0* | - | 4,830 | - |
| 1 | 196,200 | 65,260 | 0.333 |
| 2 | 149,560 | 40,202 | 0.269 |
| 3 | 111,300 | 38,197 | 0.343 |
| 4 | 91,280 | 27,603 | 0.302 |
| 5 | 59,830 | 19,662 | 0.329 |
| 6 | 35,470 | 15,209 | 0.429 |
| 7 | 36,950 | 14,604 | 0.395 |
| 8 | 29,960 | 11,237 | 0.375 |
| 9 | 24,090 | 11,889 | 0.494 |
| 10 | 20,440 | 12,341 | 0.604 |
| 11 | 20,220 | 9,586 | 0.474 |
| 12 | 19,950 | 9,991 | 0.501 |
| 13 | 18,410 | 7,554 | 0.410 |
| 14 | 15,600 | 8,426 | 0.540 |
| 15 | 12,760 | 6,712 | 0.526 |
| 16 | 652 | 243 | 0.373 |
| 17 | 10,960 | 6,409 | 0.585 |
| 18 | 11,050 | 6,446 | 0.583 |
| 19 | 9,980 | 6,327 | 0.634 |
| 20 | 14,110 | 6,830 | 0.484 |
| 21 | 6,860 | 6,128 | 0.893 |
| 22 | 4,730 | 2,658 | 0.562 |
| 23 | 5,790 | 4,462 | 0.771 |
| 24 | 6,280 | 5,203 | 0.829 |
| 25 | 2,910 | 1,540 | 0.529 |
| 26 | 5,310 | 4,091 | 0.770 |
| 27 | 5,660 | 3,706 | 0.655 |
| 28 | 4,970 | 3,049 | 0.613 |
| LGE64 | 897 | 53 | 0.059 |
| 33 | 1,650 | 122 | 0.074 |
| Z | 5,160 | 15,204 | 2.947 |
| W | 82,310 | 2 | 2.430E-05 |
| **Total** | **-** | **375,776** | **-** |

**Supplementary Table S1.** Distribution of SNPs across the chromosomes**.** *Size of the sequenced genome. * Chromosome 0 represents SNPs with no position.

| **Trait** | **SNP effect** | **SNP** | **GGA** | **Position** | ***P-value*** | **Candidate gene** |
| --- | --- | --- | --- | --- | --- | --- |
| BWG | A | rs315753164 | 1 | 141362 | 2.37e-05 | *ENSGALG00000033919* |
| BWG | A | rs312763768 | 1 | 102001483 | 3.31e-05 | *U6, ENSGALG00000037303* |
| BWG | A | rs318236242 | 1 | 73597647 | 3.75e-05 | *ENSGALG00000033533, ENSGALG00000029785* |
| BWG | A | rs317758384 | 4 | 25972470 | 1.7e-05 | *ENSGALG00000030400* |
| BWG | A | rs16185141 | 22 | 3862615 | 2.3e-05 | *ENSGALG00000030747* |
| BWG | A | rs312461641 | 22 | 4214936 | 2.8e-05 | *LRRTM4* |
| BWG | A | rs316558717 | 5 | 38947151 | 5.51e-06 | *LRRC74A* |
| BWG | A | rs14992480 | 13 | 3320197 | 6.86e-06 | *TLX2, NPM1* |
| BWG | A_sex | rs312763768 | 1 | 102001483 | 2.92e-05 | *U6, ENSGALG00000037303* |
| BWG | A_sex | rs315164331 | 3 | 23675069 | 2.92e-05 | *EML4* |
| BWG | A_sex | rs317758384 | 4 | 25972470 | 1.69e-05 | *ENSGALG00000030400* |
| BWG | A_sex | rs314678192 | 4 | 29448998 | 3.39e-05 | *ENSGALG00000009774* |
| BWG | A_sex | rs317305197 | 6 | 29287944 | 1.43e-05 | *SLC18A2, PDZD8, ENSGALG00000035332* |
| BWG | A_sex | rs14593593 | 6 | 31618521 | 3.72e-05 | *ENSGALG00000041779* |
| BWG | A_sex | rs312461641 | 22 | 4214936 | 2.71e-05 | *LRRTM4* |
| BWG | A_sex | rs313819577 | Z | 79348753 | 3.51e-05 | *ENSGALG00000034193, ZNF608* |
| BWG | A_sex | rs317588919 | 6 | 28493301 | 7.2e-07 | *ATRNL1* |
| BWG | A+D | rs315753164 | 1 | 141362 | 1.51e-05 | *ARSA* |
| BWG | A+D | rs317758384 | 4 | 25972470 | 3.75e-05 | *ENSGALG00000030400* |
| BWG | A+D | rs316558717 | 5 | 38947151 | 2.44e-05 | *LRRC74A* |
| BWG | A+D | rs316626812 | 7 | 31398092 | 1.7e-05 | *THASD7B, HNMT, ENSGALG00000033461* |
| BWG | A+D | rs316312015 | 12 | 6465550 | 3.59e-05 | *WNK2* |
| BWG | A+D | rs312461641 | 22 | 4214936 | 3.31e-05 | *LRRTM4* |
| BWG | A+D_sex | rs316498242 | 1 | 27596741 | 1.05e-05 | *ENSGALG00000027876* |
| BWG | A+D_sex | rs316423051 | 6 | 29259327 | 3.39e-05 | *PDZD8, ENSGALG00000035332* |
| BWG | A+D_sex | rs14593593 | 6 | 31618521 | 3.72e-05 | *ENSGALG00000041779* |
| BWG | A+D_sex | rs315917442 | 12 | 1173714 | 3.15e-05 | *ENSGALG00000046270* |
| BWG | A+D_sex | rs13610024 | 12 | 6127578 | 3.47e-05 | *WNT7A, PTPDC1, BARX1B, ENSGALG00000029394* |
| BWG | A+D_sex | rs313819577 | Z | 79348753 | 1.36e-05 | *ENSGALG00000034193, ZNF608* |
| BWG | A+D_sex | rs318192494 | 2 | 62758807 | 9.74e-06 | *ENSGALG00000028826, gga-mir-1759* |
| BWG | A+D_sex | rs312928242 | 12 | 8058572 | 5.02e-06 | *ERC2* |
| BWG | D | rs13610024 | 12 | 6127578 | 1.83e-05 | *WNT7A, PTPDC1, BARX1B, ENSGALG00000029394* |
| BWG | D | rs13507290 | 18 | 3730848 | 3.68e-05 | *ENSGALG00000046130* |
| FCR | A | rs15236018 | 1 | 36283561 | 1.59e-05 | *LGR5* |
| FCR | A | rs316432031 | 1 | 36706251 | 2.09e-05 | *TRHDE* |
| FCR | A | rs16083084 | 2 | 102079332 | 2.97e-05 | *PACAP* |
| FCR | A | rs314925702 | 3 | 18959711 | 3.68e-05 | *SLC30A10, EPRS* |
| FCR | A | rs14517170 | 5 | 13417627 | 1.71e-05 | *ENSGALG00000037138* |
| FCR | A | rs15801622 | 6 | 24470217 | 1.2e-05 | *SORCS3* |
| FCR | A | rs316420511 | 6 | 28472992 | 1.92e-05 | *ATRNL1* |
| FCR | A | rs13853250 | 1 | 38041526 | 5.63e-06 | *OSBPL8* |
| FCR | A_sex | rs13853250 | 1 | 38041526 | 2.87e-05 | *OSBPL8* |
| FCR | A_sex | rs15801622 | 6 | 24470217 | 1.65e-05 | *SORCS3* |
| FCR | A_sex | rs80770142 | 6 | 31514161 | 2.62e-05 | *BUB3, HMX2, HMX3* |
| FCR | A_sex | rs316420511 | 6 | 28472992 | 2.75e-06 | *ATRNL1* |
| FCR | A_sex | rs14774125 | Z | 61776792 | 1.03e-05 | *ENSGALG00000039484* |
| FCR | A_sex | rs314388497 | 6 | 29216961 | 9.29e-06 | *PDZD8* |
| FCR | A_sex | rs15936523 | 8 | 26493993 | 8.54e-06 | *FGGY* |
| FCR | A_sex | rs314893988 | 8 | 25324177 | 8.69e-06 | *PARS2* |
| FCR | A+D | rs13853250 | 1 | 38041526 | 1.78e-05 | *OSBPL8* |
| FCR | A+D | rs317318235 | 2 | 30809117 | 2.73e-06 | *RAPGEF5* |
| FCR | A+D | rs314120370 | 2 | 7676565 | 3.61e-05 | *DPP6* |
| FCR | A+D | rs14325026 | 3 | 21872815 | 1.89e-05 | *ATF3* |
| FCR | A+D | rs314377290 | 4 | 35113081 | 1.06e-05 | *FRAS1* |
| FCR | A+D | rs317423687 | 4 | 49403944 | 3.67e-05 | *TECRL* |
| FCR | A+D | rs313312956 | 12 | 8077730 | 1.4e-05 | *ERC2* |
| FCR | A+D | rs318030379 | 12 | 7469711 | 1.82e-05 | *CACNA2D3* |
| FCR | A+D | rs316312015 | 12 | 6465550 | 3.59e-05 | *WNK2* |
| FCR | A+D | rs316219360 | 1 | 195411003 | 5.89e-06 | *P2RY6* |
| FCR | A+D | rs316420587 | 1 | 194786435 | 7.07e-06 | *NUP98* |
| FCR | A+D | rs14513380 | 5 | 9756115 | 5.8e-06 | *TRIM66* |
| FCR | A+D | rs314727166 | 11 | 3384475 | 4.86e-06 | *ESRP2* |
| FCR | A+D | rs318126183 | 4 | 46851357 | 1.16e-07 | *ENSGALG00000011221* |
| FCR | A+D_sex | rs314487130 | 1 | 194771508 | 1.07e-05 | *RHOG, NUP98, PGAP2* |
| FCR | A+D_sex | rs314384561 | 1 | 92883077 | 1.1e-05 | *CHMP2B* |
| FCR | A+D_sex | rs315679231 | 1 | 76160852 | 2.29e-05 | *ENSGALG00000014252* |
| FCR | A+D_sex | rs317473017 | 2 | 23862188 | 1.84e-05 | *ENSGALG00000045286* |
| FCR | A+D_sex | rs317318235 | 2 | 30809117 | 3.55e-05 | *RAPGEF5* |
| FCR | A+D_sex | rs318126183 | 4 | 46851357 | 2.24e-05 | *ENSGALG00000011221* |
| FCR | A+D_sex | rs314377290 | 4 | 35113081 | 2.95e-05 | *FRAS1* |
| FCR | A+D_sex | rs13578747 | 6 | 22818029 | 2.05e-05 | *ENSGALG00000007803* |
| FCR | A+D_sex | rs316420511 | 6 | 28472992 | 2.25e-05 | *ATRNL1* |
| FCR | A+D_sex | rs316423051 | 6 | 29259327 | 3.49e-05 | *PDZD8, ENSGALG00000035332* |
| FCR | A+D_sex | rs314727166 | 11 | 3384475 | 3.2e-05 | *ESRP2* |
| FCR | A+D_sex | rs315862526 | 12 | 6507595 | 3.09e-05 | *WNK2* |
| FCR | A+D_sex | rs314162123 | 12 | 7387912 | 3.58e-05 | *CACNA2D3* |
| FCR | A+D_sex | rs315665292 | 13 | 7370225 | 3.45e-05 | *GABRB2* |
| FCR | A+D_sex | rs314707646 | Z | 62642824 | 3.27e-05 | *VCAN* |
| FCR | A+D_sex | rs313801640 | Z | 6165654 | 3.58e-04 | *ENSGALG00000038930* |
| FCR | A+D_sex | rs313006651 | 4 | 5995991 | 4.61e-06 | *DIAPH1* |
| FCR | A+D_sex | rs13642049 | 4 | 5496189 | 5.77e-06 | *ENSGALG00000035275* |
| FCR | A+D_sex | rs15936573 | 8 | 26507284 | 7.19e-06 | *FGGY* |
| FCR | A+D_sex | rs313312956 | 12 | 8077730 | 7.29e-06 | *ERC2* |
| FCR | A+D_sex | rs312879906 | 2 | 76462592 | 2.08e-06 | *ANKH* |
| FCR | D | rs15508269 | 1 | 173254253 | 1.02e-05 | *ENSGALG00000017062* |
| FCR | D | rs314807128 | 1 | 173564445 | 2.59e-05 | *ENSGALG00000045592* |
| FCR | D | rs314384561 | 1 | 92883077 | 3.72e-05 | *CHMP2B* |
| FCR | D | rs314724704 | 4 | 48783835 | 1.71e-05 | *ADGRL3, gga-mir-1730* |
| FCR | D | rs317370088 | 4 | 49572846 | 2.03e-05 | *SRP72* |
| FCR | D | rs317232259 | 4 | 48470791 | 2.73e-05 | *ADGRL3* |
| FCR | D | rs314350934 | 8 | 4608621 | 1.18e-05 | *ENSGALG00000033244* |
| FCR | D | rs14720659 | 9 | 15018375 | 1.21e-05 | *TNK2* |
| FCR | D | rs317503850 | 10 | 9951482 | 2.98e-05 | *SEMA6D, gga-mir-1744* |
| FCR | D | rs80591054 | 10 | 10504571 | 3.68e-05 | *GALK2, ENSGALG00000027088* |
| FCR | D | rs318030379 | 12 | 7469711 | 1.11e-05 | *CACNA2D3* |
| FCR | D | rs313312956 | 12 | 8077730 | 2.14e-05 | *ERC2* |
| FCR | D | rs315061673 | 17 | 3999541 | 2.76e-05 | *ASTN2* |
| FCR | D | rs314784590 | 21 | 3147539 | 3.23e-05 | *ENSGALG00000030881* |
| FCR | D | rs314487130 | 1 | 194771508 | 5.27e-06 | *RHOG, NUP98, PGAP2* |
| FCR | D | rs316219360 | 1 | 195411003 | 6.28e-06 | *P2RY6* |
| FCR | D | rs14325026 | 3 | 21872815 | 9.55e-06 | *ATF3* |
| FCR | D | rs314377290 | 4 | 35113081 | 6.98e-06 | *FRAS1* |
| FCR | D | rs318126183 | 4 | 46851357 | 6.04e-08 | *ENSGALG00000011221* |
| FCR | D | rs314727166 | 11 | 3384475 | 1.35e-06 | *ESRP2* |
| FI | A | rs318002020 | 1 | 57397855 | 1.23e-05 | *WEE2, SSBP1* |
| FI | A | rs13647069 | 1 | 84905430 | 1.51e-05 | *LNP1, TMEM45A* |
| FI | A | rs315522090 | 1 | 87092827 | 1.62e-05 | *BBX* |
| FI | A | rs315107540 | 1 | 112553707 | 1.71e-05 | *ENSGALG00000034279, gga-mir-6672* |
| FI | A | rs315478611 | 2 | 130411613 | 2.56e-05 | *RIMS2* |
| FI | A | rs317133732 | 2 | 145557161 | 3.81e-05 | *TRAPPC9* |
| FI | A | rs315173296 | 3 | 18410439 | 2.33e-05 | *DUSP10* |
| FI | A | rs313104423 | 3 | 13412141 | 2.79e-05 | *JAG1, gga-mir-1641* |
| FI | A | rs315381456 | 4 | 59134409 | 3.05e-05 | *UNC5C* |
| FI | A | rs317066057 | 7 | 16224389 | 1.27e-05 | *MTX2, ENSGALG00000040585* |
| FI | A | rs16580134 | 7 | 7097519 | 1.34e-05 | *PCNT* |
| FI | A | rs313616546 | 7 | 9868631 | 1.73e-05 | *HECW2* |
| FI | A | rs313139484 | 7 | 9491599 | 1.94e-05 | *SLC39A10, ENSGALG00000033329* |
| FI | A | rs316277353 | 7 | 5216065 | 2.11e-05 | *ENSGALG00000004045* |
| FI | A | rs16580847 | 7 | 8812053 | 2.6e-05 | *ENSGALG00000043345* |
| FI | A | rs15005501 | 14 | 3180770 | 2.65e-05 | *GRIFIN, ENSGALG00000033476, ENSGALG00000033476* |
| FI | A | rs316814340 | 21 | 6343049 | 1.74e-05 | *WNT4* |
| FI | A | rs313589731 | 1 | 27315930 | 8.5e-06 | *ENSGALG00000009485* |
| FI | A | rs312763768 | 1 | 102001483 | 9.5e-06 | *U6, ENSGALG00000037303* |
| FI | A | rs316682850 | 7 | 11676924 | 8.46e-06 | *CDK15* |
| FI | A | rs317640967 | 21 | 5762426 | 7.63e-06 | *DISP3, ENSGALG00000032291* |
| FI | A_sex | rs313589731 | 1 | 27315930 | 1.11e-05 | *ENSGALG00000009485* |
| FI | A_sex | rs13647069 | 1 | 84905430 | 1.65e-05 | *LNP1, TMEM45A* |
| FI | A_sex | rs312763768 | 1 | 102001483 | 3.47e-05 | *U6, ENSGALG00000037303* |
| FI | A_sex | rs316682850 | 7 | 11676924 | 2.9e-05 | *CDK15* |
| FI | A_sex | rs317203573 | 7 | 19617860 | 3.86e-05 | *SCN9A* |
| FI | A_sex | rs317640967 | 21 | 5762426 | 7.08e-06 | *DISP3, ENSGALG00000032291* |
| FI | A_sex | rs317066057 | 7 | 16224389 | 7.16e-07 | *MTX2, ENSGALG00000040585* |
| FI | A+D | rs318002020 | 1 | 57397855 | 1.23e-05 | *WEE2, SSBP1* |
| FI | A+D | rs13647069 | 1 | 84905430 | 1.51e-05 | *LNP1, TMEM45A* |
| FI | A+D | rs313254797 | 1 | 108975541 | 2.17e-05 | *RIPK4, TMPRSS2* |
| FI | A+D | rs315478611 | 2 | 130411613 | 2.94e-05 | *RIMS2* |
| FI | A+D | rs14135909 | 2 | 8603904 | 3.07e-05 | *MNX1, UBE3C* |
| FI | A+D | rs314467892 | 2 | 9263356 | 3.42e-05 | *PTPRN2* |
| FI | A+D | rs315173296 | 3 | 18410439 | 2.33e-05 | *DUSP10* |
| FI | A+D | rs313104423 | 3 | 13412141 | 2.79e-05 | *JAG1, gga-mir-1641* |
| FI | A+D | rs314348687 | 3 | 102543692 | 2.7e-05 | *APOB, LDAH* |
| FI | A+D | rs315381456 | 4 | 59134409 | 3.05e-05 | *UNC5C* |
| FI | A+D | rs317066057 | 7 | 16224389 | 3.26e-05 | *MTX2, ENSGALG00000040585* |
| FI | A+D | rs316682850 | 7 | 11676924 | 3.65e-05 | *CDK15* |
| FI | A+D | rs315030186 | 10 | 7069502 | 3.8e-05 | *ALDH1A2* |
| FI | A+D | rs316645397 | 21 | 4741412 | 2.38e-05 | *MINOS1, ENSGALG00000035240* |
| FI | A+D | rs317640967 | 21 | 5762426 | 2.46e-05 | *DISP3, ENSGALG00000032291* |
| FI | A+D | rs14846017 | 1 | 77666765 | 2.19e-06 | *CLCN1, CASP2* |
| FI | A+D | rs315107540 | 1 | 112553707 | 4.22e-06 | *ENSGALG00000034279, gga-mir-6672* |
| FI | A+D | rs313589731 | 1 | 27315930 | 8.5e-06 | *ENSGALG00000009485* |
| FI | A+D | rs312763768 | 1 | 102001483 | 9.5e-06 | *U6, ENSGALG00000037303* |
| FI | A+D | rs14069483 | 14 | 1426739 | 3.28e-06 | *TRRAP* |
| FI | A+D | rs14845471 | 1 | 77114645 | 1.2e-06 | *ZNF384* |
| FI | A+D | rs15384208 | 1 | 110917887 | 1.63e-06 | *ENSGALG00000016217* |
| FI | A+D_sex | rs313589731 | 1 | 27315930 | 1.11e-05 | *ENSGALG00000009485* |
| FI | A+D_sex | rs13647069 | 1 | 84905430 | 1.65e-05 | *LNP1, TMEM45A* |
| FI | A+D_sex | rs15384208 | 1 | 110917887 | 1.89e-05 | *ENSGALG00000016217* |
| FI | A+D_sex | rs315792569 | 1 | 77116326 | 2.91e-06 | *ZNF384* |
| FI | A+D_sex | rs315107540 | 1 | 1,13E+08 | 3.21e-05 | *ENSGALG00000034279, gga-mir-6672* |
| FI | A+D_sex | rs312763768 | 1 | 1,02E+08 | 3.47e-05 | *U6, ENSGALG00000037303* |
| FI | A+D_sex | rs318207219 | 4 | 12888606 | 1.8e-05 | *ATRX* |
| FI | A+D_sex | rs312275094 | 7 | 17014397 | 1.75e-05 | *SCRN3, SP9* |
| FI | A+D_sex | rs16590934 | 7 | 16574481 | 2.77e-05 | *LNPK* |
| FI | A+D_sex | rs316611731 | 9 | 4698401 | 2.67e-05 | *ENSGALG00000006395, ENSGALG00000037281* |
| FI | A+D_sex | rs313486854 | 9 | 20701374 | 2.72e-05 | *SERPINI1, PDCD10* |
| FI | A+D_sex | rs14069483 | 14 | 1426739 | 3.42e-05 | *TRRAP* |
| FI | A+D_sex | rs80748129 | 17 | 4324086 | 3.02e-05 | *ENSGALG00000034713, BRINP1* |
| FI | A+D_sex | rs14846017 | 1 | 77666765 | 6.16e-06 | *CLCN1, CASP2* |
| FI | A+D_sex | rs317066057 | 7 | 16224389 | 5.94e-06 | *MTX2, ENSGALG00000040585* |
| FI | A+D_sex | rs314874196 | 21 | 362229 | 9.2e-06 | *CAMTA1* |
| FI | D | rs15384208 | 1 | 110917887 | 1.49e-05 | *ENSGALG00000016217* |
| FI | D | rs317581914 | 1 | 77211449 | 2.4e-05 | *ENSGALG00000014477* |
| FI | D | rs14135909 | 2 | 8603904 | 1.98e-05 | *MNX1, UBE3C* |
| FI | D | rs314467892 | 2 | 9263356 | 2.1e-05 | *PTPRN2* |
| FI | D | rs314542965 | 2 | 40493294 | 2.53e-05 | *GPD1L, CMTM8* |
| FI | D | rs14169972 | 2 | 41645269 | 2.88e-05 | *ENSGALG00000039290, CAPN7* |
| FI | D | rs314469380 | 2 | 39800595 | 3.71e-05 | *ENSGALG00000011439, TGFBR2* |
| FI | D | rs317479100 | 9 | 1607615 | 2.6e-05 | *HS6ST1, ENSGALG00000031091* |
| FI | D | rs317904365 | 14 | 1116038 | 3.04e-05 | *TECPR1* |
| FI | D | rs316747986 | 27 | 3697594 | 1.74e-05 | *SNF8, UBE2Z, IGF2BP1* |
| FI | D | rs14069483 | 14 | 1426739 | 5.11e-07 | *TRRAP* |

**Supplementary Table S2.** SNPs associated with the three Feed Efficiency traits evaluated in broiler chickens using different SNP effects fit in the model, considering the 3 levels of significance: 5% genome-wide (2 x 10^-6^), moderate (1 x 10^-5^) and suggestive (4x10^-5^) associations.
